# Influence of the Manufacturing Method (3D Printing and Injection Molding) on Water Absorption and Mechanical and Thermal Properties of Polymer Composites Based on Poly(lactic acid)

**DOI:** 10.3390/polym16121619

**Published:** 2024-06-07

**Authors:** Paul Forbid Mukoroh, Fathi Gouda, Mikael Skrifvars, Sunil Kumar Ramamoorthy

**Affiliations:** 1 School of Engineering, Culture and Wellbeing, Arcada University of Applied Science, 00560 Helsinki, Finland; paul.forbidmukoroh@arcada.fi; 2 Department of Engineering, Faculty of Textiles, Engineering and Business, University of Borås, 501 90 Borås, Sweden; fathi.gouda@hb.se; 3 Swedish Centre for Resource Recovery, Department of Resource Recovery and Building Technology, Faculty of Textiles, Engineering and Business, University of Borås, 501 90 Borås, Sweden; mikael.skrifvars@hb.se

**Keywords:** 3D printing, injection molding, fused deposition modeling (FDM), poly(lactic acid), layer thickness, mechanical properties, thermal analysis, additive manufacturing

## Abstract

The manufacturing method influences the properties of the produced components. This work investigates the influence of manufacturing methods, such as fused deposition modeling (3D printing) and injection molding, on the water absorption and mechanical and thermal properties of the specimens produced from neat bio-based poly(lactic acid) (PLA) polymer and poly(lactic acid)/wood composites. Acrylonitrile butadiene styrene (ABS) acts as the reference material due to its low water absorption and good functional properties. The printing layer thickness is one of the factors that affects the properties of a 3D-printed specimen. The investigation includes two different layer thicknesses (0.2 mm and 0.3 mm) while maintaining uniform overall thickness of the specimens across two manufacturing methods. 3D-printed specimens absorb significantly higher amounts of water than the injection-molded specimens, and the increase in the layer thickness of the 3D-printed specimens contributes to further increased water absorption. However, the swelling due to water absorption in 3D-printed specimens decreases upon increased layer thickness. The tensile, flexural, and impact properties of all of the specimens decrease after water absorption, while the properties improve upon decreasing the layer thickness. Higher porosity upon increasing the layer thickness is the predominant factor. The results from dynamic mechanical analysis and microscopy validate the outcomes. The results from this experimental study highlight the limitations of additive manufacturing.

## 1. Introduction

Bio-based polymers, biodegradable polymers, and natural fillers, like natural fibers, cellulose, and wood, have gained enormous attention and interest due to the increasing environmental pressure on global warming. Bio-based polymers and biocomposites exhibit distinctive properties that have propelled their widespread utilization across various sectors, including the automotive, aerospace, and medical sectors. PLA is a thermoplastic and biodegradable polymer that has been used in several fields of application. It is widely considered in the medical field due to its biologically compactible nature and nontoxicity [1]. Wood is also a biodegradable and ecofriendly material like PLA, which, when milled into small pieces, can yield wood flour. This wood flour can be blended with PLA, extruded into filaments, and utilized for 3D printing [2]. Wood flour is added to PLA to reduce the cost while increasing the mechanical performance of the wood/PLA composite [3]. The development and manufacturing of polymer products play a pivotal role in the modern world. Additive manufacturing has gained a lot of space in the manufacturing industry, especially in the R&D of the above-mentioned sectors (aerospace, automotive, and medical). Fused deposition modeling (FDM)/3D printing is one of the most common additive manufacturing methods used to manufacture a component by adding material in layers. This manufacturing technique supports a wide range of materials, including polymers. The mechanical properties of a 3D-printed component will depend on several printing and manufacturing parameters. Examples of such parameters include the raster angle, the infill percentage, the printing speed, the printing temperature, the bed temperature, and the printing layer thickness [4]. The printing layer thickness is a major influencing parameter because a smaller layer height provides improved bonding and adhesion between the layers, and this increase in contact area makes the cohesion stronger and stiffens the material [5]. The printing layer thickness will also have a huge effect on the water absorption property of the printed part.

Selecting the manufacturing method for producing a part or component is a complex decision influenced by numerous factors. Across various fields of polymer application, different manufacturing methods are used to produce products tailored to meet specific design performance requirements. Over the years, 3D printing and rapid prototyping have revolutionized several industries due to their cost-effectiveness and ability to manufacture parts with complex geometries. The printing layer thickness of the component becomes a critical factor to maintain uniform properties across all of the components. Knowledge on the influence of layer thickness is also important in order to switch between manufacturing methods, as successful prototypes are often mass-produced using different manufacturing methods. Extrusion and injection molding are two of the most commonly used methods for assembly-line production in polymer industry.

Injection molding accounts for 32.5% of polymer processing processes, and it is an excellent method for mass production [6]. However, this method requires large investment, space, and high competence, and it is largely not used for prototyping [7]. The product development and prototyping are performed using simpler and cost-effective methods, such as 3D printing. 3D printing produces a three-dimensional component from a virtual model that has been designed using Computer-Aided Design (CAD) software https://www.autodesk.com/ae/solutions/cad-software (accessed on 27 May 2024). This facilitates quick and necessary changes to virtual models during the development phase, enabling the production of cost-effective prototypes quickly. This also allows us to quickly develop the existing product. However, switching between the manufacturing methods requires a thorough understanding of the shortcomings. This work intends to provide knowledge on one of the important factors, layer thickness, that affects the shift between manufacturing methods. In 3D printing, bond strength is greatly influenced by layer thickness. This crucial factor has a significant impact on the printed object’s overall quality, structural integrity, and visual appeal. Because thicker layers have less surface area available for adhesion, the bonds between them may be weaker. A structure’s overall strength increases when there are more contact sites and greater interlayer bonding due to smaller layer thickness. Layer thickness also affects other factors, like the resolution, surface finish, strength, durability, print speed, and print time.

The literature provides information about the influence of layer thickness on mechanical properties of 3D-printed polymer samples [8,9,10,11,12]. Several polymers, like ABS, PEEK, and PLA, have been used in studies. Nevertheless, the studies on 3D printed biocomposites are limited. There are also limited comparative studies between 3D printing and injection molding [11,12]. Yet, aging and water absorption of 3D-printed biocomposites are not well-explored. This work provides a comparative study of the properties of 3D-printed and injection-molded PLA, ABS, and wood/PLA composite specimens to gain knowledge on the differences in manufacturing methods through qualitative testing. Layer thickness was considered during 3D printing, while certain parameters, such as the mold temperature, cylinder temperature, injection pressure, and post-injection pressure, were optimized for injection molding. The specimens produced through 3D printing and injection molding undergo water absorption, and these specimens were tested for mechanical properties through tensile, flexural, and impact testing. Thermal analyses were performed using Differential Scanning Calorimetry (DSC) and Thermogravimetric Analysis (TGA). Dynamic mechanical analysis and microscopy were used to validate the outcomes of the above tests.

## 2. Materials and Methods

### 2.1. Materials

The filaments used for 3D printing of specimens were supplied by 3D Prima, Malmö, Sweden. Poly(lactic acid) (PrimaSelect™, Article Id: 21870, 2.85 mm diameter, Primacreator, Malmö, Sweden), wood/PLA composite with 40 wt.% wood fibers (PrimaSelect™, Article Id: 21903, 2.85 mm diameter, Primacreator), and Acrylonitrile butadiene styrene (PrimaSelect™, Article Id: 21720, 2.85 mm diameter, Primacreator) filaments were used directly without any modifications. The same filaments that were used for 3D printing were pelletized and used for injection molding. Pellets for injection molding were prepared using a pelletizer supplied by Thermo Fisher Scientific GmbH (Varicut pelletizer, Dreieich, Germany).

### 2.2. Methods

#### 2.2.1. 3D Printing

**Figure 1:**
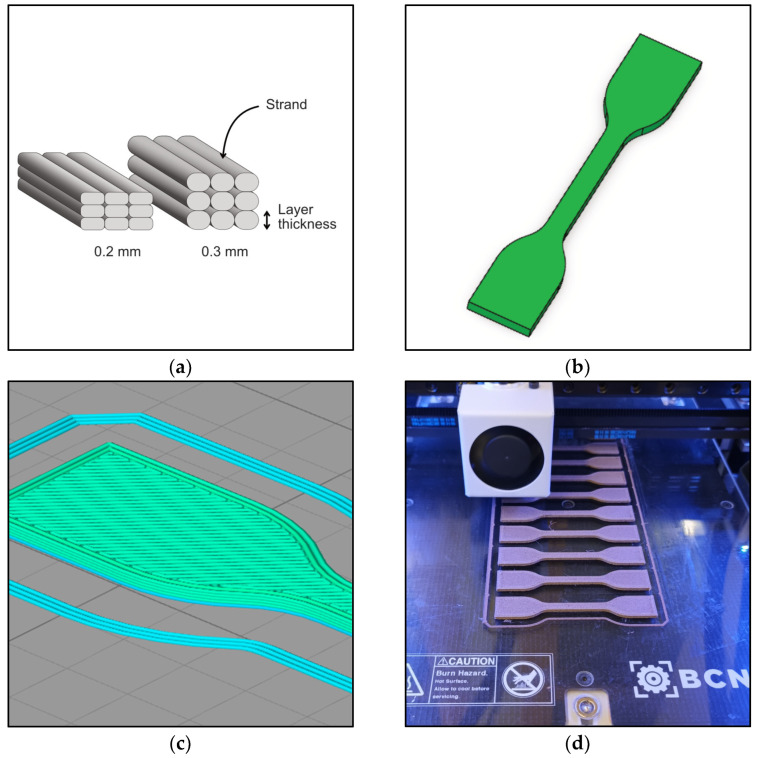
(**a**) Schematic illustration of the geometry of the 3D-printed specimens, (**b**) 3D CAD model of tensile test specimens in SolidWorks, (**c**) sliced 3D model in Simplify 3D showing layers and fill, and (**d**) 3D printing of flexural test specimens through 0.4 mm nozzle in BCN3D Sigma 3D printer.

According to ISO standards (tensile test—ISO 527/2-5A, flexural test—ISO 14125 [13], and impact test—ISO 179-1), the dog-bone and the rectangular-shaped bars with standard dimensions were designed using SolidWorks software, version 2023. The files were exported as STL files, which were then imported to the slicing software, Simplify 3D, version 5.1. The Simplify 3D parametrization software was used to generate the G-Code for 3D printing. 3D printing of tensile, flexural, and impact test specimens was performed through fused deposition modeling with the help of the BCN3D Sigma technologies dual extruder 3D printer. The characteristics of a 3D-printed component depend on the proper selection of the print settings, as this influences the component’s surface quality and mechanical properties [14]. All filaments were extruded through a 0.4 mm extruder (see Figure 1).

3D printing of PLA was performed at a temperature of 200 °C using two different layer thicknesses (0.2 mm and 0.3 mm). The two layer thicknesses chosen are close to each other to observe any change in the properties due to a small difference in layer thickness. The bed temperature was maintained at 60 °C, the percentage infill was 100%, and the print speed was 3000 mm/minute. ABS was printed with same layer thicknesses (0.2 mm and 0.3 mm) at a temperature of 250 °C. The bed temperature was 80 °C, while the percentage of infill was 100% and the print speed was 3000 mm/minute. Wood/PLA composites were printed with same layer thicknesses of 0.2 and 0.3 mm. The print temperature of 210 °C, the bed temperature of 50 °C, and the print speed of 3000 mm/minute were maintained. The print settings for all specimens have been summarized in Table 1.

#### 2.2.2. Injection Molding

All of the pellets were dried in a drying oven before injection molding. ABS pellets were dried at 80 °C for 4 h, and PLA and wood/PLA pellets were dried at 60 °C for 12 h. Pellets produced from the filaments were injection-molded using the Haake™ Minijet Pro Piston injection molding system (Thermo Fisher Scientific GmbH, Munich, Germany). For PLA, the polymer melt was injected at a temperature of 195 °C and a pressure of 550 bar, and the mold temperature was set to 25 °C. ABS melt was injected at a temperature of 230 °C and a pressure of 600 bars, whereas the mold was set at a temperature of 60 °C. Wood/PLA composite was melt-injected at a temperature of 195 °C and a pressure of 600 bars, while the mold was set at a temperature of 29 °C. Two types of specimens were manufactured, namely, the dog-bone tensile test bars according to ISO 527/2-5A and the rectangular test bars for flexural (ISO 14125, 80 × 10 × 4 mm^3^) and impact (ISO 179-1, 80 × 10 × 4 mm) testing.

### 2.3. Characterization

#### 2.3.1. Water Absorption Test

Gravimetric water absorption tests on the specimens from 3D printing and injection molding were carried out by fully immersing the specimens in water to study the influence of manufacturing methods on the hydrolytic and dimensional stability of the specimens. The tests were conducted according to the ISO 62:2008 standard [15]. All of the specimens were dried in the oven, and their weight was measured before they were immersed in the distilled water at room temperature and pressure. The water absorption of the specimens was followed for 7 days by measuring the specimens’ weight every day. The final value was noted as W_f_ for each specimen, and the percentage of water absorption (WA%) was calculated (see Equation (1)). The specimens were taken out, and the surface was carefully dried before weighing. Thicknesses of the specimens after water absorption were also measured to determine the dimensional stability of the specimens. At least fifteen measurements were taken for each specimen, and the average is reported.

(1) $$\%\;Water\;Absorption=\frac{W_{f}-W_{i}}{W_{i}}\times 100$$

#### 2.3.2. Mechanical Tests

Tensile and flexural testing of the specimens were conducted using the Tinius Olsen, Salfords, UK, H10KT Elastocon multipurpose testing machine, and the data were analyzed using Horizon software version 2020. The machine was used to measure the tensile strength, tensile modulus, elongation at fracture, flexural strength, and flexural modulus of the materials. The tensile and flexural tests of the materials were performed according to ISO 527-1 [16,17] and ISO 178 [18] standards, respectively. The tensile tests were performed by using 1 kN load cell and a 40 mm extensometer at a test speed of 5 mm/min. At least five measurements were taken for each specimen, and the average is reported. Three-point flexural tests for all specimens were performed using a 250 N load cell, and the support had a span length of 64 mm. The test speed for these tests was initially 1 mm/minute and reached a speed of 10 mm/minute when the strain was at 0.4%. The tests were performed according to well-accepted standards [19].

The impact strength of a specimen is the amount of sudden energy it absorbs before fracture [20]. The Charpy impact strength of the materials was determined by using the COMTECH impact testing machine according to ISO 179-1 [21]. The flat and un-notched specimens were tested both flatwise and endwise. Software, Meteorite 1.0, was used for the data analysis to obtain the impact energies of the specimens. The final value for the impact strength obtained is the mean value of five independent measurements.

Dynamic mechanical analysis (DMA) was carried out in a dual cantilever bending mode using the DMA Q800 TA instrument (New Castle, DE, USA). The specimens had dimensions of 50 × 10 × 3.2 mm. The tests were run at a frequency of 1 Hz and an amplitude of 15 μm. The temperature range was between 30 and 150 °C. The heating rate for all specimens was 3 °C/min. The storage modulus, which measures the energy the material stores, the loss modulus, which measures the dissipated energy, and the tan delta, which is the ratio of loss modulus to storage modulus, were recorded [22]. Experiments were performed in triplicates.

#### 2.3.3. Thermal Analysis

Thermal analysis was carried out on the specimens through two different techniques: Differential Scanning Calorimetry (DSC) and Thermogravimetric Analysis (TGA). Differential Scanning Calorimetry was conducted using DSC Q2000 TA instrument (New Castle, DE, USA). The DSC was performed to analyze the melting, the crystallization, and the glass transition temperatures of the polymers according to the ISO 11357-1 standard [23]. Specimens that weighed 5–10 mg were cut from PLA, ABS, and wood/PLA dog-bones and used for testing. All specimens underwent three thermal cycles: two heating cycles and one cooling cycle. The first heating run was performed to erase the thermal history, and this run was performed in the dynamic mode from 30 °C to 230 °C at a rate of 10 °C/minute. The specimens were then cooled down to 30 °C during the second run at 10 °C/minute. The second heating run was performed at a heating rate of 10 °C/minute from 30 °C to 230 °C. The melting temperature (Tm), crystallization temperature (Tc), and glass transition temperature (Tg) of the specimens were determined from the second and the third scans. Thermogravimetric Analysis (TGA) was performed using TGA Q800 TA instruments (New Castle, DE, USA) to determine the thermal stability of the specimens. Then, 10–20 mg was cut from the specimens and heated from 30 °C to 600 °C at a heating rate of 10 °C/min. Experiments were performed in triplicates. The tests were performed according to well-accepted standards [24].

DMA was used to measure glass transition temperatures from onset and the peaks of three different curves: storage modulus, loss modulus, and tan delta curves.

#### 2.3.4. Microscopic Analysis

The specimens’ dimensions and their internal structure were examined using Nikon polarized microscope (ECLIPSE, LV100ND POL/DS, Tokyo, Japan) at a magnification of 2.5×. The standard configuration involved setting the parameters to transmission darkfield with crossed polarizers. The morphology of the cracks, which were developed after the mechanical test on the samples, were also studied in detail using the optical microscope.

## 3. Results

### 3.1. Water Absorption Test

**Figure 2:**
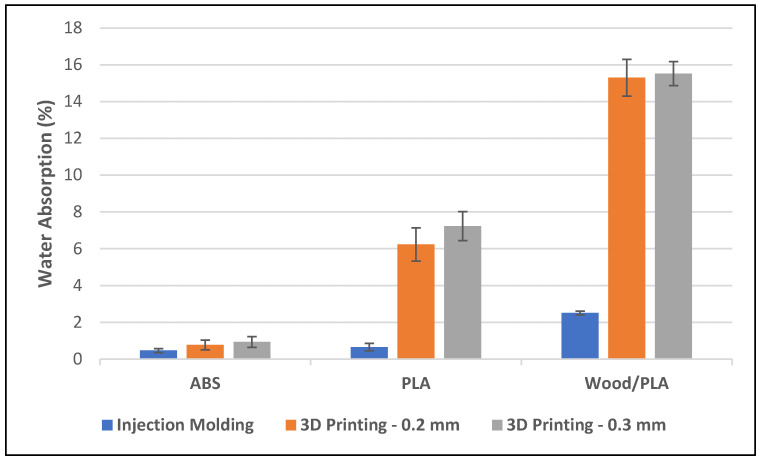
Water absorption of specimens prepared from injection molding and 3D printing.

The water absorption test demonstrated the influence of manufacturing methods on the water absorption of PLA, ABS, and wood/PLA composite. It was noticed that the manufacturing method used to produce the samples affected the water absorption and weight increase (see Figure 2). The injection-molded samples showed a lower weight increase; for instance, injection-molded PLA specimens had an average weight increase of 0.6%, and the 3D-printed PLA specimens had an average weight increase of 7.2%. This substantial increase in weight in 3D-printed specimens is due to the high level of porosity that has been induced during 3D printing. The impact of manufacturing methods was more pronounced on wood/PLA composites. When using 3D printing instead of injection molding, the water absorption of wood/PLA specimens increased to 15% from about 2%. Even the more stable polymer, ABS, had a slight increase in water absorption, demonstrating further the influence of manufacturing methods.

Increasing the layer thickness from 0.2 to 0.3 mm during 3D printing increased the water absorption. The increased water absorption can be attributed to the increased porosity within the specimens, caused by the increased printing layer thickness. The pores between the layers and the strands in the printed specimens increased upon increased printing layer thickness (see Figure 1a). Upon specimens’ immersion in water, these pores become filled with water, subsequently increasing the surface area available for water absorption into the polymer. However, the impact of layer thickness on the water absorption of specimens seems to be limited.

Wood/PLA composites are more prone to these pores due to the exposure of hydrophilic wood flour present in the strands and the layers. Hydroxyl groups in the cellulose and hemicellulose readily absorb water, as these groups make hydrogen bonds with water molecules. Increasing the surface thickness exposes more hydrophilic surface to the water molecules.

Moreover, the mechanical performances of the specimens that were immersed in water were compared with those of the dry samples (see Section 3.2).

### 3.2. Mechanical Tests

The effect of manufacturing methods, printing layer thickness, and water absorption on the mechanical properties of PLA, ABS, and wood/PLA were analyzed. The results showed that the manufacturing method used significantly impacted the mechanical properties of PLA, ABS, and wood/PLA composite specimens. It was also noticed that the mechanical properties of the specimens that were immersed in water for seven days decreased. The printing layer thickness clearly affects the mechanical properties of the specimens.

**Figure 3:**
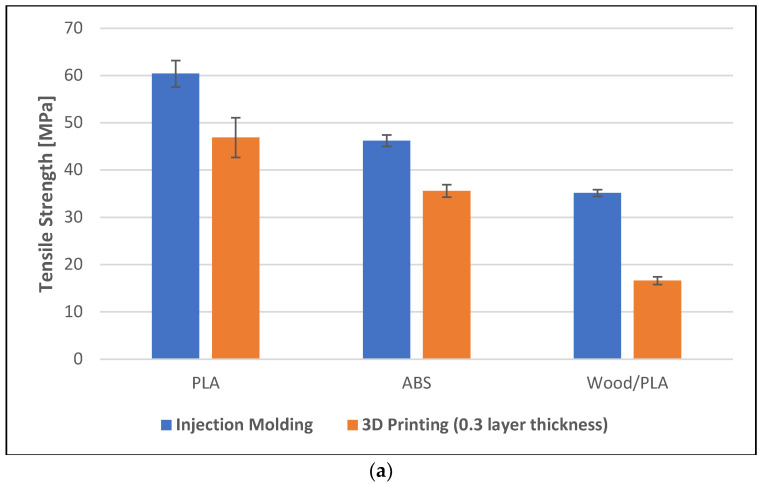

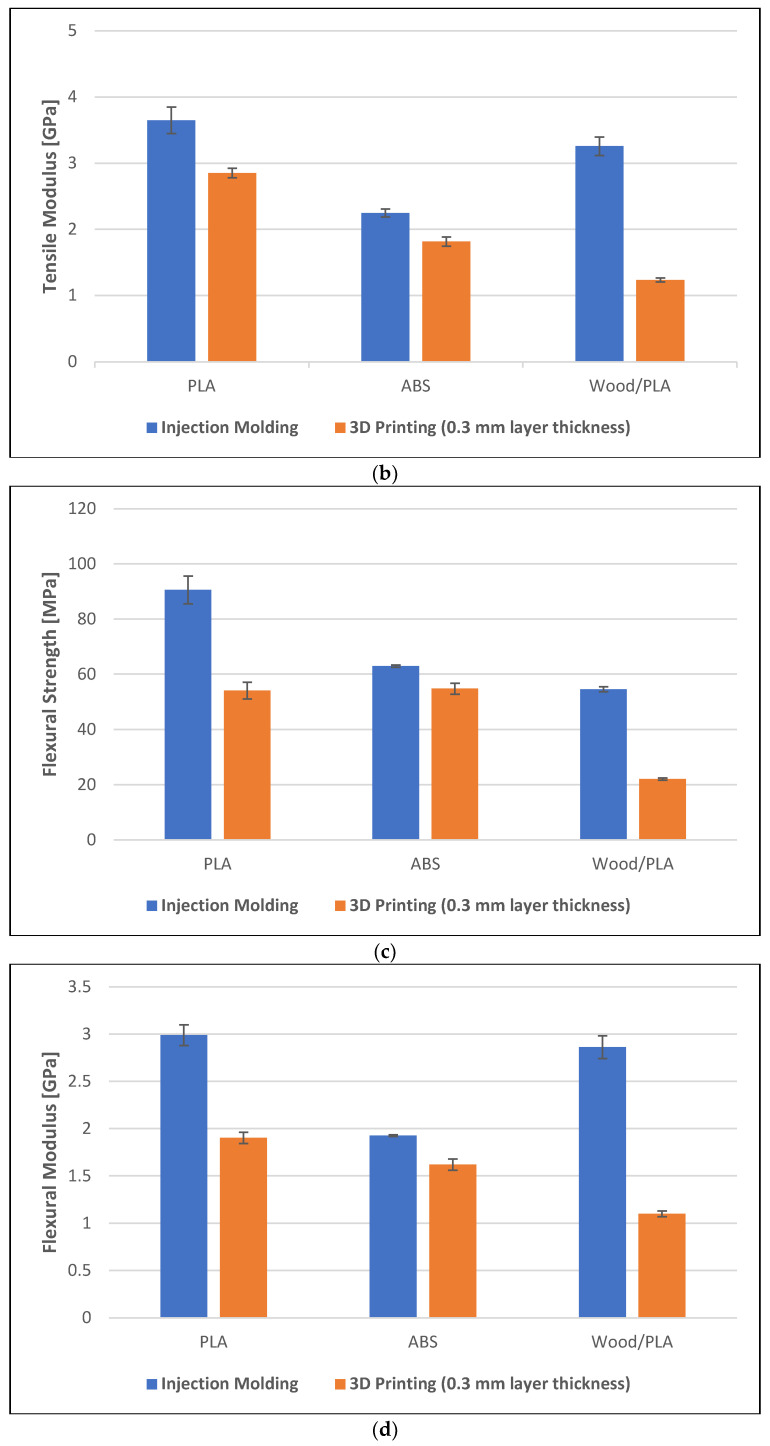
Mechanical properties of the specimens prepared using injection molding and 3D printing (0.3 mm layer thickness). (**a**) Tensile strength, (**b**) tensile modulus, (**c**) flexural strength, and (**d**) flexural modulus of the specimens.

All specimens produced through injection molding exhibited higher mechanical properties when compared to those of the 3D-printed specimens of the same polymers (see Figure 3). For example, the tensile strengths of the injection-molded PLA and 3D-printed PLA with a layer thickness of 0.3 mm were 60 MPa and 46 GPa, respectively. The tensile modulus of the injection-molded and 3D-printed (layer thickness 0.3 mm) wood/PLA was 3.2 GPa and 1.2 GPa, respectively. Similarly, the flexural strength and the flexural modulus of the specimens were significantly affected by the manufacturing method (see Figure 3c,d).

**Figure 4:**
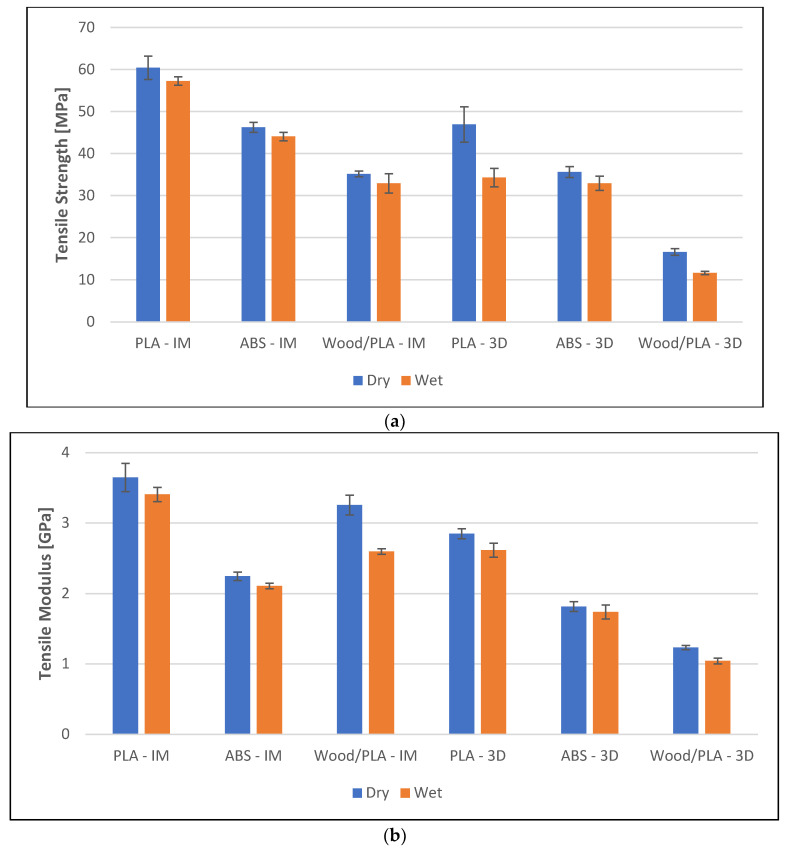

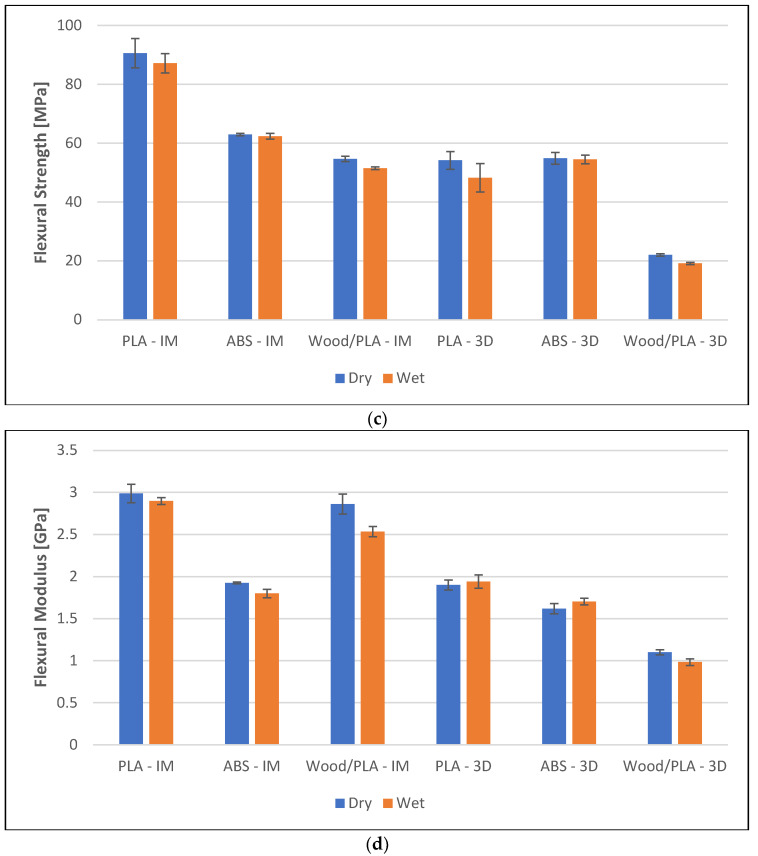
Mechanical properties of the specimens prepared using injection molding (IM) and 3D printing (3D, 0.3 mm layer thickness), both pre- and post-water immersion. (**a**) Tensile strength, (**b**) tensile modulus, (**c**) flexural strength, and (**d**) flexural modulus of the specimens.

Water absorption negatively affected the mechanical properties of the specimens (see Figure 4). However, the reduction in the tensile and flexural properties was not substantial. The limitation on the decrease of the strength and the modulus was a result of fairly stable polymers and the specimens being immersed in water for a short duration. Nevertheless, it is well-known that the aging process involving higher temperature and humidity severely affects the mechanical properties of PLA and its composites [25]. The discussion of the aging process falls beyond the scope of this work’s focus. The results show that the manufacturing method amplifies the reduction in the mechanical properties.

**Figure 5:**
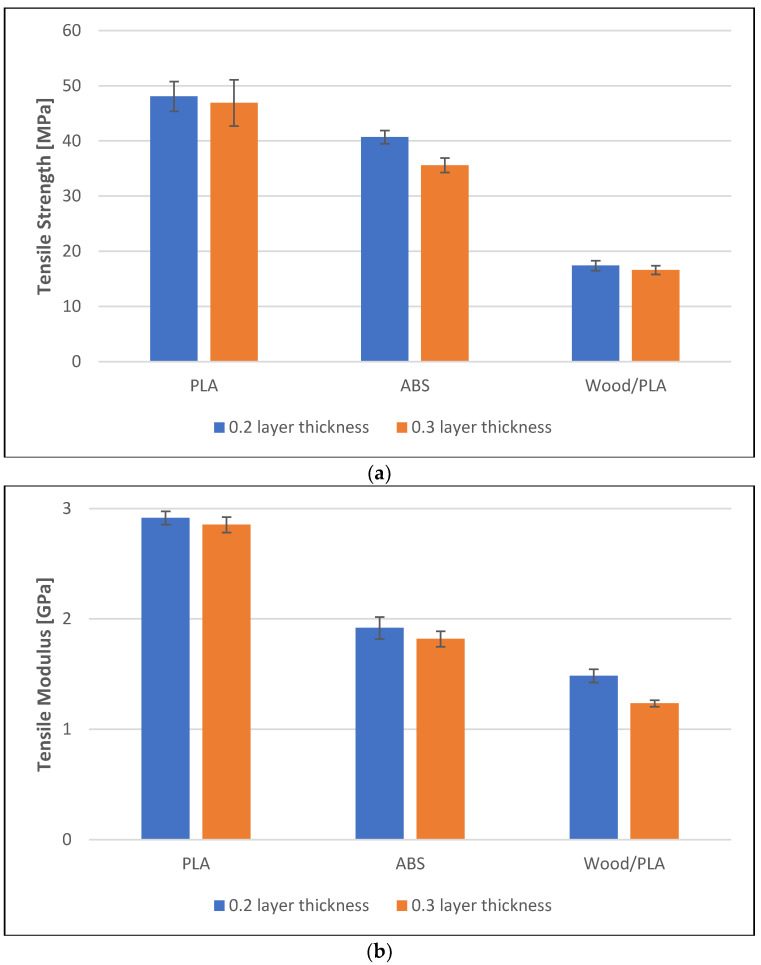

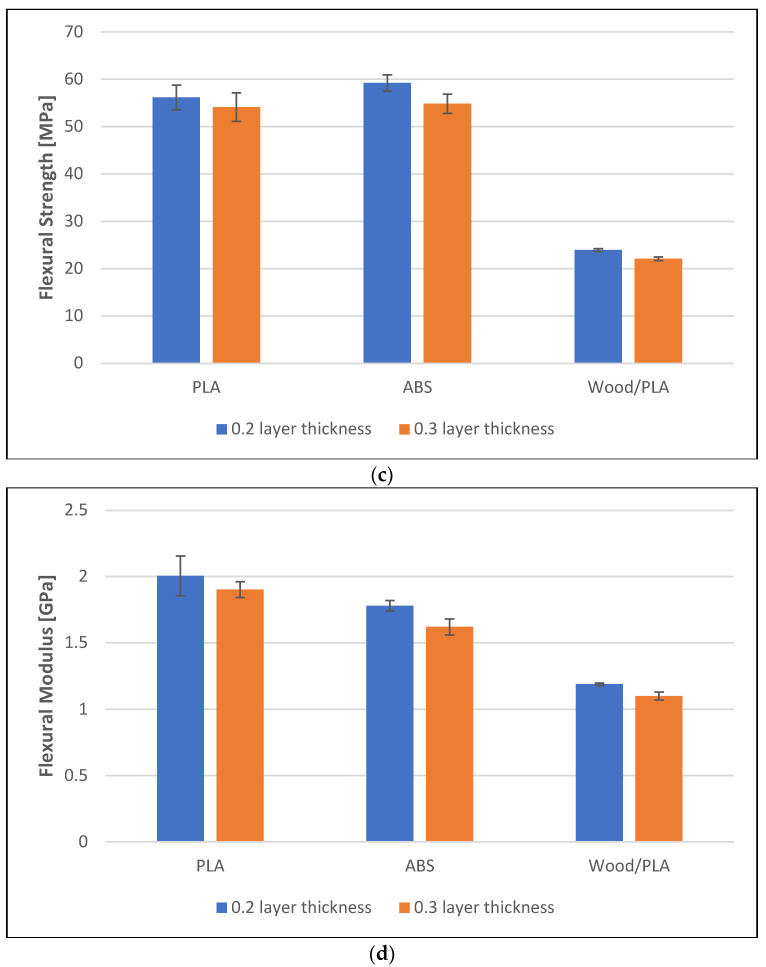
Mechanical properties of the specimens prepared using 3D printing with two different layer thicknesses, 0.2 mm and 0.3 mm. (**a**) Tensile strength, (**b**) tensile modulus, (**c**) flexural strength, and (**d**) flexural modulus of the specimens.

The specimens that had a printing layer thickness of 0.2 mm had higher tensile strength compared to the samples that had a printing layer thickness of 0.3 mm. For instance, the tensile strength of dry ABS specimens that had printing layer thicknesses of 0.2 mm and 0.3 mm were 40 MPa and 35 MPa, respectively. PLA and wood/PLA specimens also had a slight decrease in the tensile strength. The trend observed in the tensile modulus, flexural strength, and flexural modulus of the specimens was consistent with the tensile strength, as they were affected by the change in the layer thickness (see Figure 5). This decrease in mechanical properties with the increase in printing layer thickness is due to the presence of pores and the presence of unfilled gaps within the specimens. These pores weaken the adhesion between the layers that leads to a weak bond strength. The pores in the 3D-printed samples weakened the structure of the specimens and made them vulnerable to failure during impact, tensile loading, and flexural loading due to the decrease in their internal resistance. Porosity causes stress concentrations, which are mostly located around the edges of the voids. These stress concentrations cause a reduction in the mechanical properties of the material. The increase in the printing layer thickness also reduces the time required for the specimens to cool, and this affects the adhesion between the layers, hence reducing the mechanical properties [26]. The mechanical properties were further affected when the specimens were immersed in the water (see Table S1).

Table 2 shows the Charpy impact strength of the specimens, and the results show the influence of the manufacturing methods, the printing layer thickness in 3D printing, and the water absorption. The manufacturing method has the most significant influence on the specimens’ impact strength. The edgewise impact strength of the injection-molded PLA specimen measured 67 kJ/m^2^, which decreased by 80% when the manufacturing method was changed. A similar observation was noted in wood/PLA specimens. The change in the layer thickness from 0.2 to 0.3 mm had a limited effect on the impact properties. However, the water absorption of the specimens had a more significant effect on the impact strength.

Storage and loss moduli from the DMA curves show that the printing layer thickness and manufacturing methods affect the mechanical properties of the specimens (see Table 3). This falls in line with the other mechanical test results. As mentioned earlier, there is a direct correlation between a smaller layer height and increased bond strength, as demonstrated by the results from DMA. The results also show that the damping was severely restricted upon the addition of wood flour.

The results of the mechanical tests fall in line with the results from similar work [2,3,4,5]. Research has shown an evident link between layer thickness and the mechanical properties of 3D-printed wood/PLA. Thicker layers consistently result in poorer mechanical performance. Thicker layers weaken the 3D-printed parts. This happens because thicker layers create larger gaps between them, increasing the overall porosity within the specimen. More pores mean less solid material, leading to a decrease in overall strength and modulus of the printed specimen. In simpler terms, the research has found that using thinner layers packs more material into the same volume, resulting in stronger and stiffer printed parts [2,3,4,5].

### 3.3. Thermal Analysis

DSC results confirmed that the manufacturing methods do not have significant effect on the glass transition temperature (Tg), the crystallization temperature (Tc), or the melting temperature (Tm) of PLA and their composites (Table 4). This was expected, as the polymer processing temperatures are similar in both manufacturing methods and the molecular chains of the polymer after the injection molding and the 3D printing will be affected minimally. The effect of water absorption on thermal properties was also minor, as the key thermal transitions (glass transition, crystallization, and melting) remained unaffected by water absorption. However, the addition of wood flour to the PLA affected the glass transition and the crystallization. This is due to the restricted movements of polymer chains in the presence of reinforcement particles.

Table 5 shows the glass transition temperatures obtained from the DMA’s storage moduli, loss moduli, and tan δ curves. In general, the Tg obtained from DMA was higher than the Tg from DSC. This is expected due to the sensitivity of the instruments [27]. However, the trend observed in DMA confirms the results from DSC; the addition of reinforcement particles affected the Tg of the specimens, and the manufacturing method has minimal effect on Tg.

**Figure 6:**
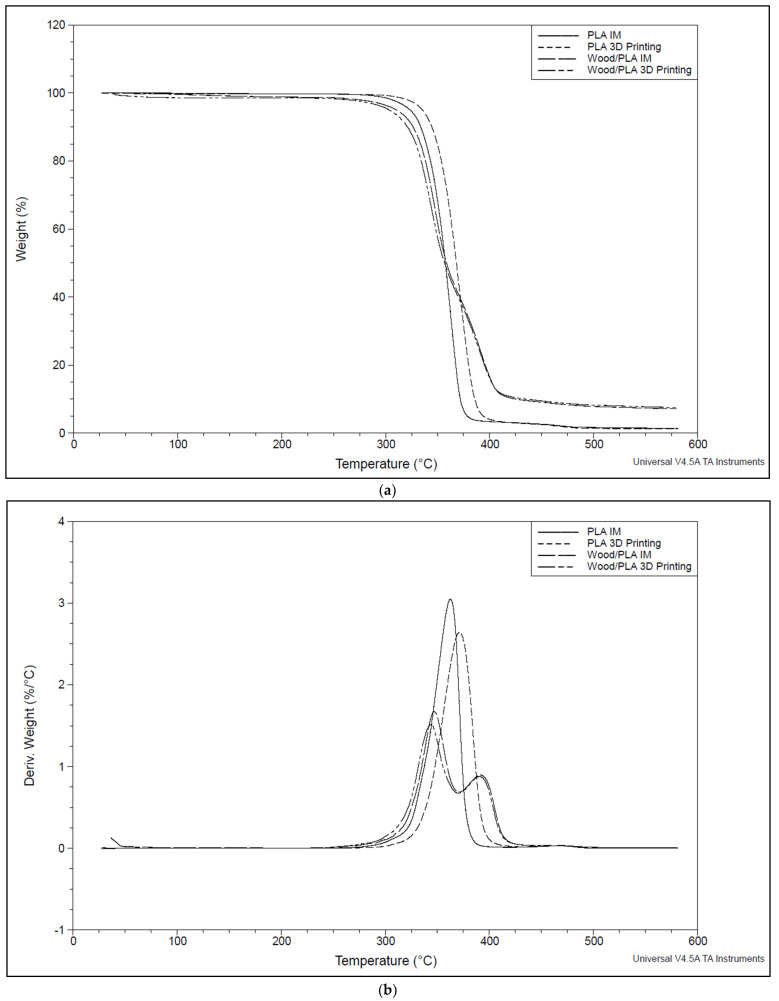

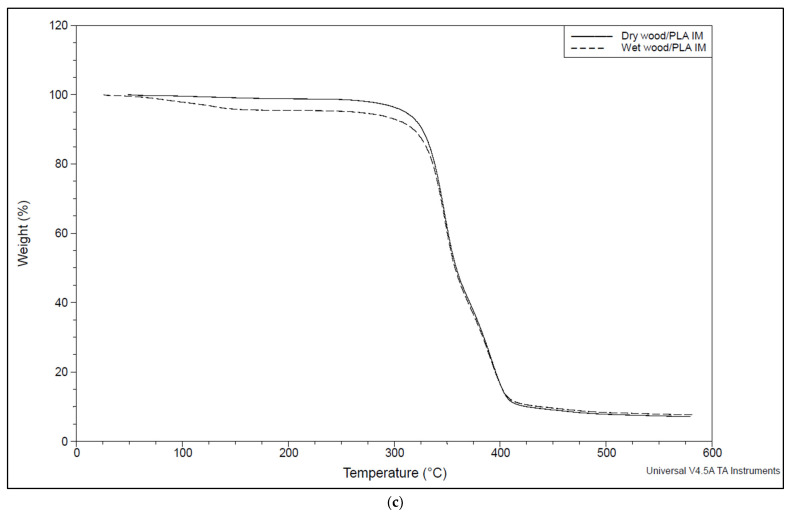
TGA graphs of PLA and wood/PLA. (**a**) Comparison of impact of manufacturing technologies on thermal degradation; (**b**) one-step degradation of PLA and two-step degradation of wood/PLA; (**c**) comparison of impact of wet and dry samples on thermal degradation.

TGA curves show that the polymers underwent a single-stage decomposition, and the composite (wood/PLA) underwent a multistage decomposition within the temperature range of 30 and 600 °C. Manufacturing methods did not affect the degradation temperature of the specimens, as expected. However, the water absorption of the specimens slightly lowered the degradation initiation temperature (see Figure 6).

### 3.4. Microscopic Analysis

**Figure 7:**
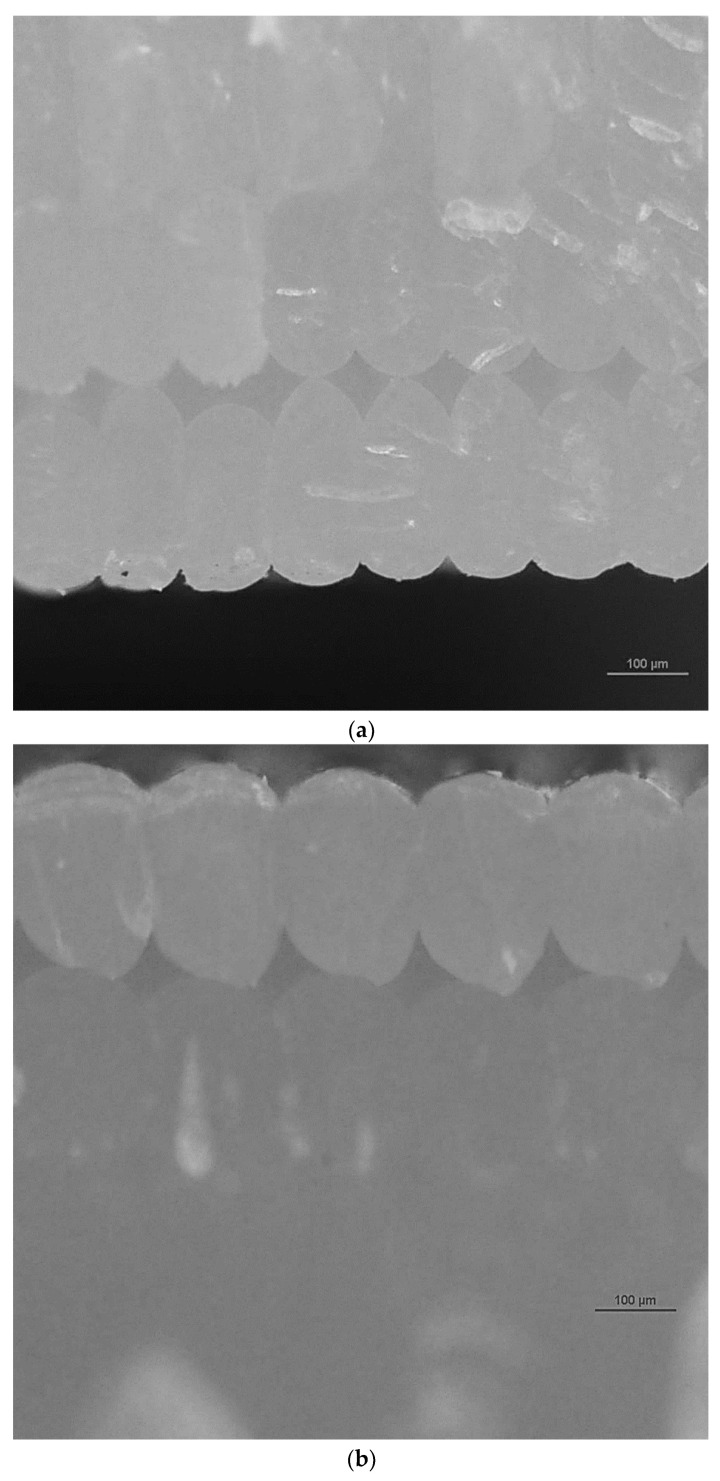

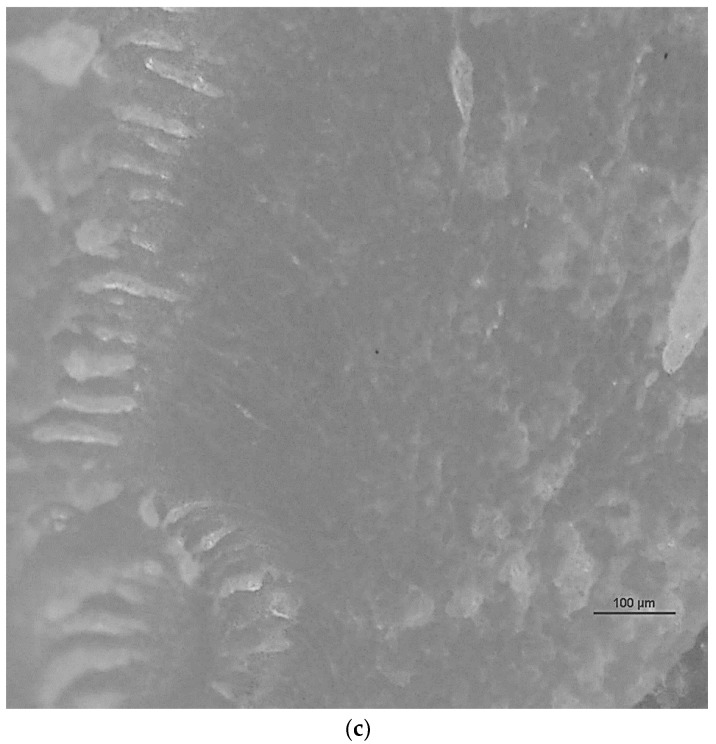
Fractured surface’s microscopic images of PLA: (**a**) 0.2 mm layer thickness, 3D-printed specimen, (**b**) 0.3 mm layer thickness, 3D printed-specimen, and (**c**) injection-molded specimen.

**Figure 8:**
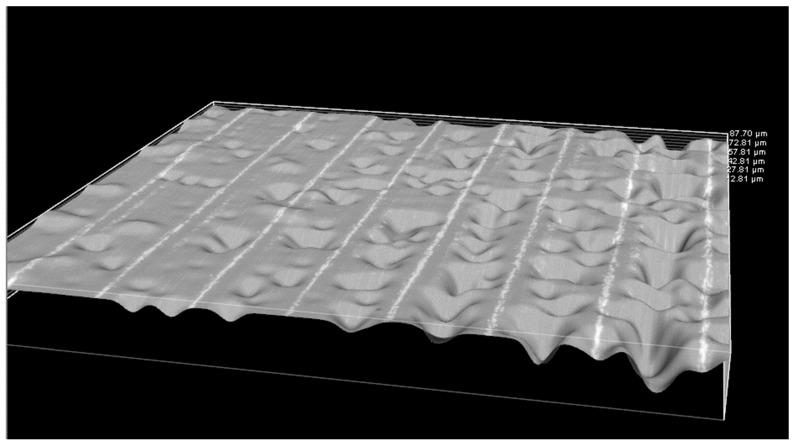
Three-dimensional image of the cross-section of the unfractured surface of the 3D-printed PLA specimen with a layer thickness of 0.2 mm.

Figure 7 shows microscopic images illustrating fractured surface parts post-testing. These images were captured from the fractured areas of the specimens during the test, revealing the failure mechanisms, the layers in 3D-printed specimens, and the pores in the specimens. The images show that the pore size increases upon increasing the layer thickness in 3D printing, while the injection-molded samples did not show any visible pores. The results fall in line with the results obtained from mechanical testing. Furthermore, Figure 8 shows the three-dimensional image of the cross-section of the unfractured surface of the 3D-printed PLA specimen, clearly revealing the layers and the pores.

**Figure 9:**
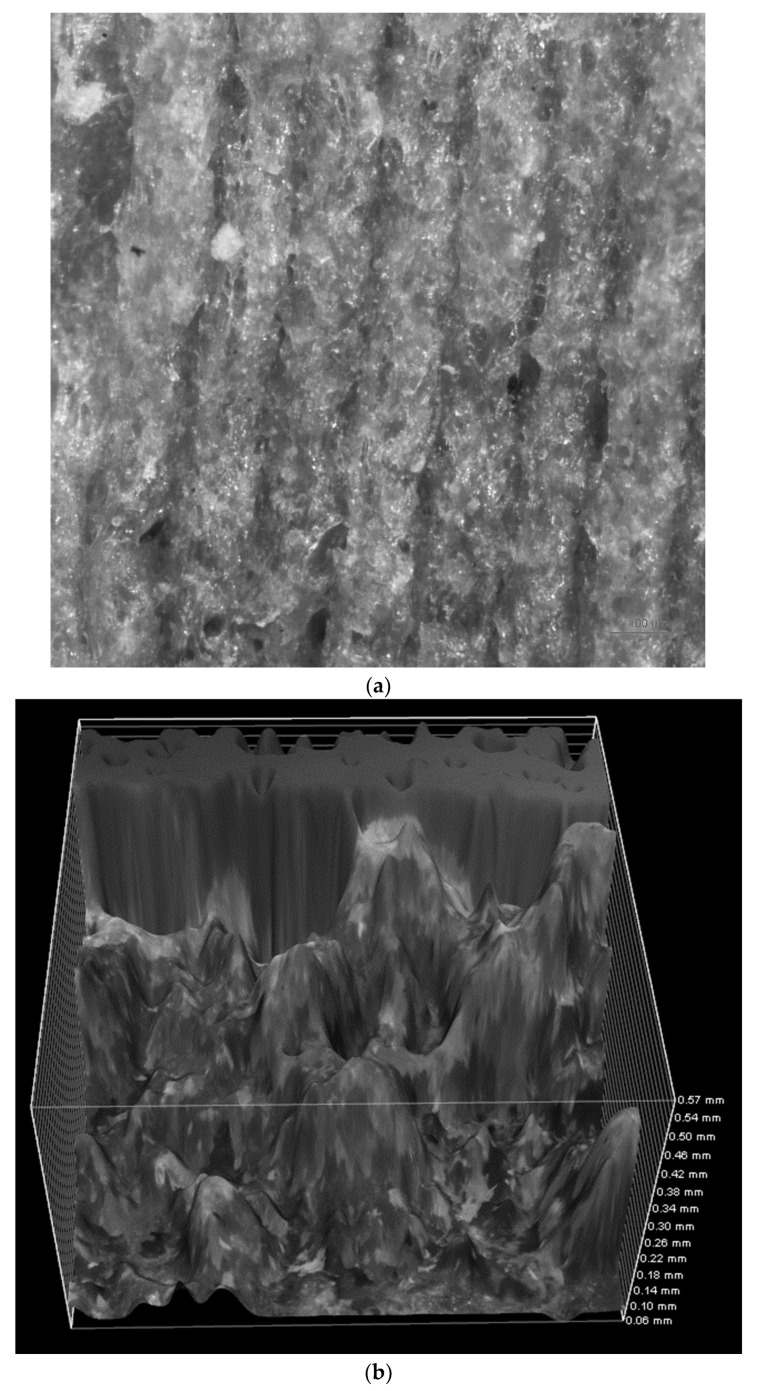

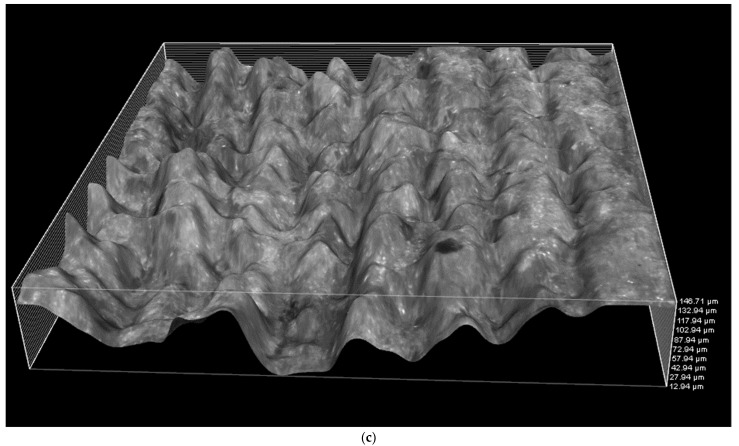
Microscopic images of 3D-printed wood/PLA specimens with a 0.2 layer thickness. (**a**) Cross-section of dry, unfractured surface; (**b**) three-dimensional image of cross-section of dry, unfractured surface; and (**c**) three-dimensional image of cross-section of wet, unfractured surface.

Figure 9 shows the microscopic images of the wood/PLA composites. The layers were clearly seen on the 3D-printed specimens, and the water absorption compromised the dimensional stability of the specimens by increasing the pore size, the layer size, and the entire thickness of the sample. This effect was not as clear while using the injection molding method for specimen preparation.

The distortions that appear on images of 3D-printed specimens were due to non-uniform layer height, warping, and layer shifting. Inconsistent layer height occurred when the thicknesses of the layers were not uniform. This variation could also appear due to the inconsistent extrusion rate, which makes the layer height differ. Warping caused the corner edges of the printed samples to curl downwards, caused by uneven cooling and poor temperature gradients.

## 4. Discussion

A well-established polymer processing technique, injection molding, is used for the mass production of various plastic products, while recent advancements in additive manufacturing help in mass customization and rapid prototyping. The results from this work show that the specimens produced from injection molding and 3D printing vary in terms of mechanical and morphological properties. Furthermore, the parameter (layer thickness) set for the 3D printing also affected certain properties.

Water absorption of the specimens produced from these manufacturing techniques was affected by the material properties and the porosity. The water absorption of the injection-molded specimens was primarily due to the material properties, while the water absorption of the 3D-printed specimens was due to the combination of the intrinsic porosity of the 3D printing and the material properties. Water absorption in injection-molded specimens is most likely due to the gradual absorption of water from the surface to the core. Increasing the specimens’ surface contact with water in 3D printing increased the water absorption. Additionally, increasing the layer thickness in 3D printing increased the water absorption further.

Mechanical properties, such as tensile and flexural properties, were affected by the manufacturing methods. Injection-molded specimens had higher density and better mechanical properties than 3D-printed specimens due to solid specimens resulting in reduced failure sites. In the case of 3D-printed specimens, the porosity reduced the density and increased the number of failure sites within the specimens. The manufacturing method of the specimens had a huge influence on the impact strength of the specimens. The water absorption of the specimens and the layer thickness in 3D-printed specimens also affected the mechanical properties. These results from the above mechanical testing correlated closely with the results obtained from DMA.

Thermal properties of the specimens were not affected significantly upon changing the manufacturing method. This was anticipated, as the polymer processing temperatures in both methods were similar, and there will be minimal impact on the polymer’s molecular chains following injection molding and 3D printing. Water absorption had a negligible impact on thermal characteristics, as well. Nevertheless, the glass transition and crystallization were impacted by the addition of wood flour to PLA. This results from the polymer chains’ limited mobility in the presence of reinforcing particles. TGA results conclude that there is no significant difference in thermal degradation of specimens produced through two different methods.

Morphological analysis shows the increase in the porosity upon increasing the layer thickness. The different layers in 3D printing and their fusing points were also noticed. Layer distortion in 3D-printed specimens was noticed upon water absorption, affecting the dimensional stability of the specimens. The results fall in line with the mechanical results obtained in this work.

The layer thickness in 3D printing plays a crucial role in influencing the bond strength. This critical parameter significantly impacts the overall quality, structural integrity, and visual appearance of the printed object. Thicker layers may result in weaker bonds between layers due to reduced surface area for adhesion. Smaller layer thickness allows for more contact points and increased interlayer bonding, enhancing the overall strength of the structure. It is more intuitive to have a large difference between layer thicknesses, which would show large difference in the results. However, we like to show that the smallest difference in layer thickness causes a change in the properties.

Several industries can benefit from the above results. Electronics and aerospace are continuously adopting 3D printing technologies and capabilities to lower the weight, produce intricate parts, and automize sections of the production line. These results can show the limitations of this manufacturing method. Medical applications are adopting 3D printing due to its mass customization and design capabilities. The results show that the properties of specimens produced using 3D printing could bring certain restrictions.

## 5. Conclusions

- Mechanical properties, such as tensile, flexural, and impact properties, of the specimens produced from two different manufacturing methods, such as 3D printing and injection molding, vary.
- Manufacturing methods do not have a significant effect on thermal properties.
- Morphological analysis shows the increase in the porosity upon increasing the layer thickness.
- Water absorption of the specimens was affected by the manufacturing methods.
- Layer distortion in 3D-printed specimens was noticed upon water absorption, affecting the dimensional stability and mechanical properties of the specimens.

## Figures and Tables

**Table 1:** 3D printer settings of polymers and composites.

Settings	PLA	ABS	Wood/PLA
Nozzle diameter, mm	0.4	0.4	0.4
Infill/%	100	100	100
Layer thickness, mm	0.2, 0.3	0.2, 0.3	0.2, 0.3
Print speed, mm/s	5	5	5
Nozzle temperature, °C	200	250	210
Bed temperature, °C	60	80	60

**Table 2:** Charpy impact strength of the specimens tested both edgewise and flatwise.

	Manufacturing Method	Specimen	Impact Strength, Edgewise (kJ/m^2^)	Impact Strength, Flatwise (kJ/m^2^)
DRY	IM	PLA	67.7 (22.8)	15.7 (2.5)
		ABS	19.9 (1.6)	84.6 (0.1)
		Wood/PLA	25.2 (2.5)	17.2 (0.7)
	3D Printing (0.2)	PLA	12.3 (2.8)	11.1 (2.0)
		ABS	25.5 (2.9)	37.9 (2.5)
		Wood/PLA	8.3 (0.4)	7.8 (0.1)
	3D Printing (0.3)	PLA	13.5 (1.3)	9.9 (1.3)
		ABS	26.7 (3.4)	27.9 (1.3)
		Wood/PLA	9.5 (1.3)	7.3 (0.6)
WET	IM	PLA	50.1 (20.1)	15.9 (1.1)
		ABS	15.4 (1.8)	83.3 (0.1)
		Wood/PLA	22.3 (0.9)	17.2 (1.7)
	3D Printing (0.2)	PLA	13.6 (2.4)	12.7 (2.8)
		ABS	22.2 (4.9)	29.3 (3.6)
		Wood/PLA	9.9 (0.8)	9.2 (0.4)
	3D Printing (0.3)	PLA	12.5 (0.4)	9.5 (1.5)
		ABS	25.2 (1.9)	23.7 (2.6)
		Wood/PLA	7.3 (1.2)	7.2 (0.8)

The number within the parentheses denotes the standard deviation of the mean. IM denotes injection molding.

**Table 3:** Storage moduli (E′), loss moduli (E″), and damping factors of injection-molded and 3D-printed specimens.

Manufacturing Method	Specimen	Storage Modulus, E′ Max (MPa)	Loss Modulus, E″ Max (MPa)	Damping Factor Tan δ (×10^−2^)
IM	PLA	2321	432	18.6
	ABS	1236	172	13.9
	Wood/PLA	1682	220	13.0
3D Printing (0.2)	PLA	2365	440	18.6
	ABS	1450	195	13.4
	Wood/PLA	1044	128	12.3
3D Printing (0.3)	PLA	1906	326	17.1
	ABS	1247	163	13.1
	Wood/PLA	928	117	12.6

**Table 4:** Glass transition, crystallization, and melting-temperatures of PLA and their composites obtained from DSC.

	Manufacturing Method	Specimen	Tg °C	Tc °C	Tm °C
DRY	IM	PLA	54.0	109.7	159.4
		Wood/PLA	57.3	86.7	148.8
	3D Printing	PLA	53.9	109.7	159.9
		Wood/PLA	57.1	86.7	149.2
WET	IM	PLA	53.3	109.3	159.2
		Wood/PLA	56.4	86.9	149.0
	3D Printing	PLA	53.8	109.3	159.2
		Wood/PLA	57.7	86.7	149.5

Tg—glass transition temperature; Tc—crystallization temperature; Tm—melting temperature.

**Table 5:** Glass transition temperatures of PLA and their composites obtained from DMA.

Manufacturing Method	Specimen	Tg from E′ °C	Tg from E″ °C	Tg from tan δ °C
IM	PLA	54.3	54.7	61.3
	Wood/PLA	61.3	61.9	66.7
3D Printing (0.2)	PLA	53.5	53.9	60.7
	Wood/PLA	60.8	61.1	67.0
3D Printing (0.3)	PLA	53.6	53.6	61.1
	Wood/PLA	61.2	61.5	68.0

## Data Availability

Data are available within the article.

## Supplementary Materials

The following supporting information can be downloaded at: https://www.mdpi.com/article/10.3390/polym16121619/s1, Table S1: Tensile and flexural properties of all of the tested specimens in all tested conditions.
