# Supplementary material for: Influence of the Manufacturing Method (3D Printing and Injection Molding) on Water Absorption and Mechanical and Thermal Properties of Polymer Composites Based on Poly(lactic acid)
Source: Polymers (Basel). 2024 Jun 7;16(12):1619. doi: 10.3390/polym16121619 (PMC11207301; doi:10.3390/polym16121619)
Supplement: Supplementary file 1 [file polymers-16-01619-s001.zip › polymers-2992469-supplementary.pdf]

Table S1: Tensile and flexural properties of all the tested specimens in all tested conditions.

		Specimen	Tensile modulus (GPa)	Standard Deviation	Tensile strength (MPa)	Standard Deviation	Strain at fracture (%)	Standard Deviation	Flexural strength (MPa)	Standard Deviation	Flexural modulus (GPa)	Standard Deviation
**DRY**	**IM**	**PLA**	3.6	0.2	60.4	2.8	2.3	0.2	90.5	5	2.9	0.10
		**ABS**	2.2	0.06	46.2	1.2	8.2	0.1	62.9	0.4	1.9	0.01
		**Wood/PLA**	3.2	0.1	35.1	0.7	4.1	0.5	54.6	0.9	2.8	0.10
	**3D Printing (0.2)**	**PLA**	2.9	0.06	48.1	2.7	4.5	1.3	56.1	2.6	2.0	0.10
		**ABS**	1.9	0.1	40.7	1.2	5.6	0.8	59.2	1.7	1.7	0.04
		**Wood/PLA**	1.4	0.06	17.4	0.9	3.4	0.3	23.9	0.3	1.1	0.01
	**3D Printing (0.3)**	**PLA**	2.8	0.07	46.9	4.2	3.0	0.3	54.1	3	1.9	0.06
		**ABS**	1.8	0.07	35.6	1.3	5.7	1.1	54.8	2	1.6	0.06
		**Wood/PLA**	1.2	0.03	16.6	0.8	3.5	0.6	22.0	0.4	1.1	0.03
**WET**	**IM**	**PLA**	3.4	0.1	57.2	1.0	6.1	3.9	87.1	3.3	2.8	0.04
		**ABS**	2.1	0.04	44.0	1.0	16.8	2.8	62.3	1	1.8	0.05
		**Wood/PLA**	2.5	0.04	32.9	2.3	4.5	0.6	51.5	0.4	2.5	0.06
	**3D Printing (0.2)**	**PLA**	2.8	0.07	41.2	3.9	5.8	3.3	46.1	3.5	1.9	0.10
		**ABS**	1.8	0.1	40.5	1.0	6.9	2.3	55.6	1.9	1.8	0.03
		**Wood/PLA**	1.1	0.01	12.7	0.8	3.6	0.5	19.1	0.8	1.0	0.03
	**3D Printing (0.3)**	**PLA**	2.6	0.1	34.3	2.2	4.4	0.7	48.2	4.8	1.9	0.08
		**ABS**	1.7	0.1	32.9	1.7	3.9	0.7	54.4	1.5	1.7	0.04
		**Wood/PLA**	1.0	0.04	11.6	0.4	11.4	0.6	19.1	0.4	0.9	0.04
